# *In Vitro* Control of Uropathogenic Microorganisms with the Ethanolic Extract from the Leaves of* Cochlospermum regium* (Schrank) Pilger

**DOI:** 10.1155/2017/4687154

**Published:** 2017-12-11

**Authors:** Danny Ellen Meireles Leme, Allan Belarmino Rodrigues, Adriana Araújo de Almeida-Apolonio, Fabiana Gomes da Silva Dantas, Melyssa Fernanda Norman Negri, Terezinha Inez Estivalet Svidzinski, Jonas da Silva Mota, Claudia Andrea Lima Cardoso, Kelly Mari Pires de Oliveira

**Affiliations:** ^1^Faculty of Exact Sciences and Technology, Federal University of Grande Dourados, Dourados, MS, Brazil; ^2^Faculty of Medicine, Federal University of Mato Grosso do Sul, Campo Grande, MS, Brazil; ^3^Faculty of Health Sciences, Federal University of Grande Dourados, Dourados, MS, Brazil; ^4^Department of Clinical Analysis and Biomedicine, State University of Maringá, Maringá, PR, Brazil; ^5^Course of Chemistry, State University of Mato Grosso do Sul, Dourados, MS, Brazil; ^6^Faculty of Biological and Environmental Science, Federal University of Grande Dourados, Dourados, MS, Brazil

## Abstract

The roots of* Cochlospermum regium*, popularly known as “algodãozinho-do-cerrado,” are used for the treatment of genitourinary infections. However, the removal of their subterranean structures results in the death of the plant, and the use of the leaves becomes a viable alternative. Therefore, the antimicrobial activity of* Cochlospermum regium* leaf's ethanolic extract and its action on the biofilm formation of microorganisms associated with urinary infection were evaluated. The total phenolic compounds, flavoids, and tannins were quantified using the reagents Folin-Ciocalteu, aluminum chloride, and vanillin, respectively. The antimicrobial activity was evaluated by the broth microdilution method and the effect of the extract in the biofilm treatment was measured by the* drop plate* method. Cytotoxicity was evaluated by the method based on the reduction of MTS and the mutagenicity by the Ames test. The ethanolic extract of* C. regium* leaves presented 87.4 mg/EQ of flavonoids, 167.2 mg/EAG of total phenolic compounds, and 21.7 mg/ECA of condensed tannins. It presented reduction of the biofilm formation for* E. coli* and* C. tropicalis* and antimicrobial action of 1 mg/mL and 0.5 mg/mL, respectively. The extract showed no cytotoxicity and mutagenicity at the concentrations tested. This study demonstrated that* C. regium *leaves are a viable option for the treatment of genitourinary infections and for the species preservation.

## 1. Introduction

Urinary tract infection (UTI) is a public health problem that affects millions of people every year [1]. It is defined as the colonization of pathogenic microorganisms that affect the urinary system tissues causing infection [2]. Urinary infection of bacterial origin is most often caused by enteric Gram-negative bacteria, with* Escherichia coli* being the most predominant microorganism [3, 4]. Opportunistic microorganisms such as yeast are also considered uropathogens, and the genus* Candida* has been reported as one of the most important [5]. Among yeasts of this genus,* Candida tropicalis* is among the most isolated ones in patients diagnosed with urinary infection [6].

A serious nosocomial problem currently faced in relation to these microorganisms is the formation of biofilm, which is characterized as microbial communities with complex organizational structure that may be present in hospital equipment and instruments causing or aggravating infections [7, 8].

Since ancient times, medicinal plants are alternative resources used by the population for therapeutic purposes [9]. It is estimated that approximately 90% of people have already used natural medicine for the primary relief of some discomfort caused by diseases [10]. The World Health Organization (WHO) [11] encourages research regarding the use of plants, with the aim of ensuring that the therapeutic properties present in these natural products can be applied to prevent/treat diseases without damaging health.

*Cochlospermum regium* (Schrank) Pilger is a shrub of the family Bixaceae Kunth, popularly known as “algodãozinho-do-campo” or “algodãozinho-do-cerrado” [12]. According to sellers of medicinal herbs,* C. regium *roots and xylopods are among the most sought-after medicinal plants and are indicated for the therapeutic treatment of uterine, ovarian, and prostate inflammation and infection [13]. In addition to the antimicrobial action, the roots and xylopods also present analgesic, antidematogenic and anti-inflammatory activity [14, 15]. These biological activities may be related to the presence of phenolic compounds, such as ellagic acid, gallic acid, dihydrokaempferol-3-O-*β*-glucopyranoside and dihydrokaempferol-3-O-*β*-glucopyranoside and dihydrokaempferol-3-O-*β*-(6′′-galloyl)-glucopyranoside, pinoresinol, excel sine, and two triacil benzenes known as Cochlospermines A and B [14]. In relation to the leaves of* C. regium*, several substances have already been identified and isolated from their essential oil showing the presence of 96.87% sesquiterpenes, being 18.73%  *β*-copaen-4-*α*-ol and 12.67% of viridiflorol [16]. However, in the literature there are still no reports of antimicrobial activity with* C. regium* leaves.

Much used because of its subterranean parts,* C. regium* is among the priority species for conservation, due to the fact that the removal of its roots causes the destruction of the bush [17] and, for this reason, the number of researches on the processes of germination and* in vitro* preservation increases [18, 19]. In this sense, the use of the leaves has been proposed in order to minimize the impacts on the plant, since previous studies report the presence of the same compounds as in the root. Thus, the aim of this work was to evaluate the antimicrobial activity and action on the biofilm formation of microorganisms associated with urinary infection, from the ethanolic extract of* Cochlospermum regium* leaves.

## 2. Material and Methods

The leaves of* Cochlospermum regium* were collected at Santa Madalena Farm (S 22° 13′ 41.8′′/W 054° 49′ 58.4′′), Dourados, MS. The exsiccate was identified by Dr. Zefa Valdivina Pereira and deposited (DDMS 5001) in the Herbarium of the Faculdade de Ciências Biológicas e Ambientais from Universidade Federal da Grande Dourados, Dourados, MS, Brazil.

The dry and crushed vegetable material (200 g) was mixed in 1000 mL of 95% absolute ethyl alcohol (Dynamics Contemporary Chemistry Ltd., Diadema, BRA) and maintained at 25°C for 72 h with shaking about every 12 h. The solutions were filtered and evaporated (Rotaevapor R-215) at 35°C until complete solvent volatilization. The obtained plant extract was lyophilized (E-C MicroModulyo coupled to valuPump VLP80 Savant vacuum pump).

### 2.1. Quantification of Phenolic Compounds of the Ethanolic Extract of Cochlospermum Regium Leaves

Tests were performed to track the following classes of compounds: organic acids [20], steroids and triterpenes [21], flavonoids [21], total phenolic compounds, condensed tannins [21], and alkaloids [21, 22]. Among these classes flavonoids, total phenolic compounds, and condensed tannins were quantified.

The concentration of flavonoids was determined using the method described by Lin and Tang [23] and the results were expressed as milligram equivalents of quercetin (mg/EQ) per gram of the ethanolic extract. The concentration of total phenolic compounds was determined by the method of Djeridane et al. [24] and the results were expressed as milligram equivalents of gallic acid (mg/EGA) per gram of ethanolic extract. The concentration of condensed tannins was determined by the method proposed by Broadhurst and Jones [25] and adapted by Agostini-costa et al. [26]. The results were expressed as milligram equivalent of catechin (mg/ECA) per gram of extract.

### 2.2. Minimal Inhibitory Concentration and Minimum Bactericidal/Fungicidal Concentration

The Minimal Inhibitory Concentration (MIC) of the extract was determined by the broth microdilution method, according to the guidelines of the* Clinical and Laboratory Standards Institute* [27, 28], with adaptations for natural products. Microorganisms from the American Type Culture Collection (ATCC, Rockville, MD, USA),* Escherichia coli* (ATCC 25922) and* Candida tropicalis* (ATCC 750), and three clinical isolates from each of these species were tested (Laboratory of Applied Microbiology, University of Grande Dourados, Dourados).

Bacteria were cultured on Tryptone Soy agar (TSA, Himedia, Mumbai, India) at 37°C for 24 h and the inoculum concentration was adjusted to 1.5 × 10^8^ CFU/mL using a wavelength of 625 nm in a spectrophotometer (Visible Digital Microprocessor Q898DRM-QUIMIS ISO 8001). The yeasts were cultured on Sabouraud Dextrose agar (SDA, Himedia, Mumbai, IND) at 35°C for 48 h and the inoculum concentration adjusted to 2.5 × 10^6^ CFU/mL at the 530 nm wavelength.

The extract was dissolved in dimethyl sulfoxide (DMSO, Sigma-Aldrich) and subjected to successive dilutions (1 : 2) in 96 well plates with Müeller Hinton broth (MHB, Himedia, Mumbai, India) for bacteria and RPMI 1640 broth (Sigma-Aldrich, São Paulo, Brazil) for the yeasts, obtaining concentrations from 1 mg/mL to 0.0019 mg/mL. The antibiotic ampicillin (AMP, Sigma-Aldrich, São Paulo, Brazil) and antifungal fluconazole (FLU, Sigma-Aldrich, São Paulo, BRA) were used to control the assay.

For bacteria, in addition to visual reading, the minimum inhibitory concentration (MIC) was determined by adding to the wells 50 *μ*L of 0.1% solution of triphenyltetrazolium chloride (TTC, Vetec, Sigma-Aldrich, São Paulo, Brazil) for 30 min. The yeast reading was performed visually. CIM was considered as the lowest concentration of the extract in which the microorganisms did not show visible growth [29]. The test was performed in duplicate at three different times.

To determine the minimum bactericidal concentration (MBC) and minimum fungicidal concentration (MFC), a 10 *μ*L aliquot of all wells from the microplate was removed and plated on a Müeller Hinton agar (Himedia, Mumbai, India) for bacteria and Sabouraud Dextrose agar (SDA, Himedia, Mumbai, India) for yeast. MBC and MFC were defined as the lowest concentration in which there was no colony growth [30].

Microbial activity was determined according to the parameters established by Kuete [31], significant activity (MIC < 0.1 mg/mL), moderate activity (0.1 mg/mL ≤ MIC ≤ 0.625 mg/mL), and low activity (MIC > 0.625 mg/mL).

### 2.3. Antimicrobial Activity in Biofilm

The microorganisms* Escherichia coli* (ATCC 25922) and* Candida tropicalis* (ATCC 750) were tested.

To prepare the inoculum,* E. coli* was resuspended in 25 mL of Tryptone Soy Broth (TSB, Himedia, Mumbai, India) at 37°C and* C. tropicalis* in Sabouraud Dextrose broth (SDB, Himedia, Mumbai, India) at 35°C; both resuspensions were kept overnight under 80 rpm orbital shaking. After incubation, the cells were washed three times with 25 ml of 0.1 M phosphate buffered saline (PBS) pH 7.0 and centrifuged at 10,000*g* for 5 min at 4°C. Subsequently, they were adjusted in the Neubauer counting chamber at a concentration of 2.48 × 10^8^ CFU/mL of bacteria and 5 × 10^5^ CFU/mL of yeast.

To determine the efficacy of the extract in the treatment of biofilm, the assays were carried out in 96-well flat bottom microplates (Nunclon, Delta, Nunc A/S, Roskilde, Denmark), according to the methodology proposed by Costa et al. [32], with some modifications. For biofilm formation, 200 *μ*L of preadjusted inoculum was added to the microplate wells and incubated at 37°C for 24 h for bacteria and at 35°C for 48 h for yeast under orbital shaking at 80 rpm.

After biofilm formation, the wells were washed three times with 200 *μ*l of PBS and then treated with different concentrations of the extract (16 mg/mL, 8 mg/mL, 4 mg/mL, 2 mg/mL, and 1 mg/mL). After this step, the wells were aspirated and washed three times with 200 *μ*l of PBS for removal of the poorly adhered cells. For quantification of the biofilm formed, the wells were scraped and the obtained suspensions were serially diluted with 10 replicates for each concentration and plated by the* Drop plate* method in Müeller Hinton agar (MHA, Himedia, Mumbai, India) for the bacterium and Sabouraud Dextrose agar (SDA, Himedia, Mumbai, India) for yeast.

The assays were performed in triplicate and, as control of biofilm formation, the inoculum and its corresponding culture medium were added. Subsequently, the colony forming units (CFU) were counted and the results were converted to log⁡10.

### 2.4. Cytotoxicity Assay

The cytotoxicity of the ethanolic extract from the leaves of* C. regium* was evaluated using HeLa cell lines (Henrietta Lacks, cervical adenocarcinoma cell line) and VERO (lineage established from African green monkey kidney cells,* Cercopithecus aethiops*). HeLa cells were cultured in Dulbecco's Modified Eagle's medium (DMEM, Gibco, Waltham, USA) with 10% fetal bovine serum (FBS, Gibco, Waltham, USA) and VERO cells in RPMI 1640 medium (Sigma-Aldrich, USA). Cells set at 2 × 10^5^ cells/mL were added to the 96-well microplates (Kasvi, Curitiba, Brazil) and incubated at 37°C under 5% CO_2_ for 24 h. After the incubation period, the adhered cells were washed and treated with different concentrations of the extract (0.5 mg/mL, 0.15 mg/mL, and 0.05 mg/mL) and incubated under the same conditions. For growth control (white) the culture medium and the cell suspension were used.

Cell viability was evaluated based on the reduction of MTS (3-[4,5-dimethylthiazol-2-yl]-5-[3-carboxymethoxyphenyl]-2-[4-sulfophenyl]-2H-tetrazolium) (Promega, Madison Charter Township, USA). After 3 h of incubation at 37°C, the formazan absorbance was measured at 490 nm wavelength, using an ASYS microplate reader (Biochrom, Holliston, USA). Optical density (OD) values were converted to percent cell viability by dividing the absorbance value of the sample by the absorbance of the blank and multiplying the result by 100. The assays were performed in triplicate at three different times [33, 34].

### 2.5. Mutagenicity Assay

The mutagenic potential of the ethanolic extract of* C. regium* leaves was evaluated by the microsuspension method developed by Maron and Ames [35] with some modifications proposed by Kado et al. [36]. The assays were performed with strains of* Salmonella* Typhimurium TA97a, TA98, TA100, and TA102 in the presence and absence of metabolic activation.

The overnight bacterial suspension was concentrated at 2 × 10^9^ bacteria/mL by centrifugation and then resuspended in sodium phosphate buffer solution (pH 7.4, 0.2 mM). 50 *μ*L of adjusted inoculum was added to the tubes containing 50 *μ*L of phosphate buffer solution (pH 7.4, 0.2 mM) or S9 mixture (Molotoxic Molecular Toxicology Inc., USA) and 5 *μ*L of the different concentrations of the extract (2 mg/mL, 1 mg/mL, 0.5 mg/mL, 0.25 mg/mL, and 0.125 mg/mL). The tubes were preincubated for 90 min at 37°C.

Following this time, 2 mL of top agar [0.6% (w/v) sodium chloride, 0.6% (w/v) agar-agar (Difco, USA), 0.5 mM D-biotin (Sigma-Aldrich, USA), 0.5% L-histidine (w/v) (Sigma-Aldrich, USA)] was added to the solution and the tubes were homogenized and poured into glycated minimum agar (GMA) plates [1.5% (w/v) agar-agar (Difco, USA), 20% glucose solution (w/v) (Vetec, Brazil), and 2% (v/v) Vogel-Bonner solution]. The number of revertant colonies per* his*+ plate was measured after 66 h at 37°C. The assays were performed in triplicate.

The positive controls used in the assays without metabolic activation were 4-nitro-o-phenylenediamine (NPD, Sigma-Aldrich, USA) (0.5 *μ*g/plate) for TA97a and TA98 and sodium azide (Sigma-Aldrich, USA) (2.5 *μ*g/plate) for TA100 and mitomycin C (Sigma-Aldrich, USA) (0.5 *μ*g/plate) for TA102. In the metabolic activation assays the 2-aminoanthracene compound (2-AA, Sigma-Aldrich, USA) (0.625 *μ*g/plate) was used for all strains. Negative controls were performed with the sample solvent, DMSO (5 *μ*L/plate).

### 2.6. Statistical Analysis

Data on inhibition of preformed biofilm and cytotoxicity were analysed using GraphPad Prism 5.0 statistical software (GraphPad Software, Inc., USA). Ames test data were analyzed without the statistical program Salanal (US Environmental Protection Agency, Monitoring Systems Laboratory, USA, version 1.0, Research Research Triangle Institute, RTP, USA).

## 3. Results

The ethanolic extract of the leaves (EEF) of* C. regium* indicated the presence of organic acids, steroids and triterpenes, flavonoids, total phenolic compounds and condensed tannins, and absence of alkaloids. A total of 87.4 ± 0.9 mg/EQ of flavonoids, 167.2 ± 2.7 mg/EAG of total phenolic compounds, and 21.7 ± 0.1 mg/ECA of condensed tannins were quantified.

It presented antimicrobial activity against microorganisms associated with urinary infection. The ethanolic extract of the leaves of C. regium presented a low antimicrobial activity (1 mg/mL) for* E. coli* and a moderate activity (0.5 mg/mL)* C. tropicalis* (Table 1).

**Figure 1:**
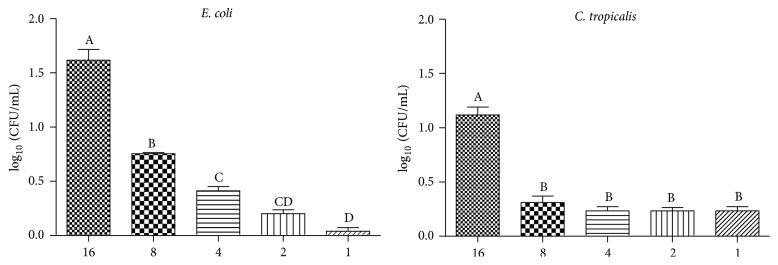
Reduction of CFU/mL (log10) number of* Escherichia coli* and* Candida tropicalis* biofilm treated with different concentrations of the ethanolic extract of* Cochlospermum regium* leaves in mg/mL. Different letters represent statistically significant differences found (*p* < 0.05).

At the concentrations of 16 mg/mL and 8 mg/mL, the* C. regium* EEF reduced the preformed* E. coli* biofilm and at the concentration of 16 mg/mL the* C. tropicalis* biofilm was also reduced (Figure 1).

**Figure 2:**
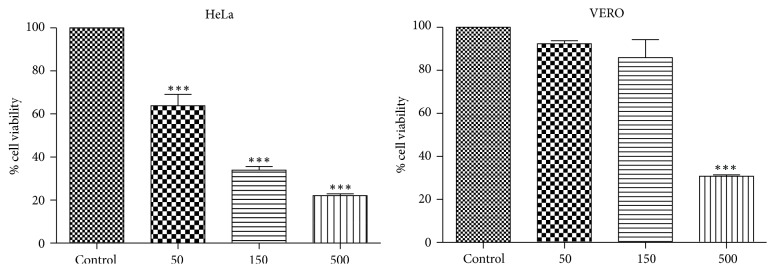
Cell viability of HeLa and VERO cells treated with ethanolic extract of* C. regium* for 24 hours. ^*∗∗∗*^*P* < 0.001 (ANOVA).

The* C. regium* EEF showed 60% of cell viability for HeLa and 92% for VERO at a concentration of 0.50 mg/mL (Figure 2).

The EEF of* C. regium* did not present a mutagenic potential, in the absence and presence of the metabolic activation system (S9), at all tested concentrations and for all evaluated strains of* Salmonella* Typhimurium (Tables 2 and 3).

## 4. Discussion

The EEF of* C. regium* presented organic acids, steroids and triterpenes, flavonoids, total phenolic compounds, condensed tannins, and absence of alkaloids. These results corroborate with Vasconcelos Filho et al. [37] in a study carried out with the leaves, stem, and root of* C. regium* and report that phenolic compounds are among the most commonly found compounds in these parts of the plant, among them tannins and absence of alkaloids in their leaves.

According to Goulas et al. [38], phytochemicals may exhibit antimicrobial activity. Authors report that the terpenes and tannins found in the leaves of* C. regium* have activity against fungi and bacteria [39, 40]. In addition, the EEF of* C. regium* also presents as major compounds the phenolic compounds and flavonoids, which have been reported in the literature as antimicrobials, being responsible for the rupture of the cell membrane of microorganisms and inhibition of nucleic acid synthesis and energetic metabolism [41, 42]. The leaves of* C. regium* presented an antimicrobial action against* E. coli*, establishing a correlation with the popular use, since this bacterium is responsible for 80–90% of cases of urinary infection [43]. Another species of the genus* Cochlospermum* [44] has also been associated with activity against* E. coli*, reiterating that this genus has antimicrobial properties.

For* Candida tropicalis*, the leaf extract presented antifungal action at the concentration of 0.5 mg/mL; this data is in agreement with the results of Inácio et al. [45], who demonstrated antifungal action of the* C. regium* root, this way, indicating that the leaves can be used in substitution to the popular use of the roots helping in the preservation of this species. Rodrigues and de Carvalho [46] report that medicinal species which have the highest risk of extinction are those whose parts used for the preparation of the medicines are roots, stem, or bark of the stem.

*Candida *is a genus of yeast fungi, which are present in the mucosa, skin, and gastrointestinal tract and make up the vaginal and urethra microbiota. They are considered commensal and opportunistic, and if there is an imbalance due to low immunity, they may prevail the microbiota and become pathogenic [8]. Authors report that* Candida tropicalis* is among the most isolated microorganisms in patients with urinary tract infection [6].

The* C. regium* EEF reduced the biofilm formed by* E. coli* and* C. tropicalis*. Adhesion and biofilm formation are virulence factors that promote the increase of antimicrobial resistance and the permanence of these microorganisms in the genitourinary tract. The reduction of the formed biofilm is relevant data, since, when adhered, these microorganisms become less susceptible to the antimicrobial activity.

The use of medicinal plants in the treatment of diseases requires information that ensures absence of toxicity [47]. The* C. regium* EEF did not show cytotoxicity against VERO cells and it evidenced a potential antiproliferative activity against HeLa cells. Another study has also shown absence of cytotoxicity in* C. regium* [48].

The* C. regium* EEF did not show mutagenicity by the Ames test. The assay was performed in the presence and absence of S9 microsomal fraction which simulates an exogenous metabolization process similar to the hepatic metabolism. The sample is considered mutagenic when at least one of the strains has a mutagenicity index greater than or equal to 2 and a dose response relationship between the concentrations tested [49]. Nunes and de Carvalho [50] evaluated the mutagenic potential of the* C. regium* root extract with* Drosophila melanogaster* germ cells and found that the extract also does not present mutagenicity by this model.

## 5. Conclusion

The study showed that the* Cochlospermum regium* EEF showed antimicrobial activity and reduction of biofilm formation in relation to uropathogens, such as* Escherichia coli* and* Candida tropicalis*; in addition, it does not present cytotoxicity and mutagenicity by the evaluated tests and concentrations. In this way, the present study enables the use of the leaves, since its use minimizes the impacts caused by the removal of roots and* Xylopodia*.

## Figures and Tables

**Table 1:** Minimum inhibitory concentration, minimal bactericidal concentration, and minimum fungicidal concentration in mg/mL of the ethanolic extract of *Cochlospermum regium* leaves against microorganisms associated with urinary infection.

Microorganisms	MIC	MBC	MFC	AMP	FLU
*Escherichia coli *(ATCC 25922)	1	—	*∗*	0.032	*∗*
*Escherichia coli* (1)	1	—	*∗*	>0.016	*∗*
*Escherichia coli* (2)	1	—	*∗*	>0.016	*∗*
*Escherichia coli *(3)	1	—	*∗*	>0.016	*∗*
*Candida tropicalis *(ATCC 750)	0.5	*∗*	—	*∗*	0.001
*Candida tropicalis *(1)	0.5	*∗*	—	*∗*	0.001
*Candida tropicalis *(2)	0.5	*∗*	—	*∗*	0.001
*Candida tropicalis *(3)	0.5	*∗*	0.5	*∗*	0.001

MIC: minimum inhibitory concentration; MBC: minimum bactericidal concentration; MFC: minimum fungicide concentration; (—): absence of antimicrobial activity; (*∗*): not tested; AMP: ampicillin; FLU: fluconazole.

**Table 2:** Mutagenic potential expressed by mean and standard deviation of revertant colonies per plate and mutagenicity index in the absence of metabolic activation of the ethanolic extract of *Cochlospermum regium* leaves.

Concentration (mg/plate)	TA97a		TA98		TA100		TA102	
	Mea/SD	MI	Mea/SD	MI	Mea/SD	MI	Mea/SD	MI
0.0	117 ± 9	1	45 ± 9	1	164 ± 15	1	364 ± 35	1
0.125	99 ± 21	0.84	32 ± 1	0.70	87 ± 8	0.53	402 ± 86	1.10
0.25	112 ± 12	0.95	33 ± 3	0.72	113 ± 37	0.69	442 ± 13	1.21
0.5	138 ± 10	1.18	26 ± 1	0.52	89 ± 8	0.54	360 ± 19	0.98
1	107 ± 13	0.91	26 ± 1	0.57	116 ± 34	0.70	372 ± 29	1.01
2	133 ± 25	1.14	26 ± 1	0.57	85 ± 34	0.52	432 ± 14^*∗*^	1.18

Mean/SD: mean of revertant colonies and standard deviation; MI: mutagenicity index; 0.0: negative control (dimethyl sulfoxide, DMSO). ^*∗*^*P* < 0.05 (ANOVA).

**Table 3:** Mutagenic potential expressed by the mean and standard deviation of revertant colonies per plate and mutagenicity index in the presence of metabolic activation of *Cochlospermum regium* leaves ethanolic extract.

Concentration (mg/plate)	TA97a		TA98		TA100		TA102	
	Mea/SD	MI	Mea/SD	MI	Mea/SD	MI	Mea/SD	MI
0.0	139 ± 8	1	22 ± 5	1	142 ± 5	1	398 ± 9	1
0.125	155 ± 10	1.11	37 ± 6	1.67	117 ± 22	0.82	400 ± 17	1.00
0.25	145 ± 7	1.03	29 ± 13	1.31	134 ± 38	0.94	437 ± 18	1.09
0.5	141 ± 12	1.01	31 ± 3	1.41	182 ± 20	1.28	509 ± 13	1.27
1	138 ± 8	0.99	28 ± 6	1.28	237 ± 16	1.66	420 ± 20	1.05
2	131 ± 13	0.93	30 ± 7	1.35	237 ± 15^*∗∗*^	1.66	528 ± 4	1.32

Mea/SD: mean of revertant colonies and standard deviation; MI: mutagenicity index; 0.0: negative control (dimethyl sulfoxide, DMSO). ^*∗∗*^*P* < 0.01 (ANOVA).
